# The era of “Infectious Diseases+” has arrived: multi-disciplinary integration in pediatric infectious disease prevention and control

**DOI:** 10.3389/fped.2026.1659176

**Published:** 2026-03-10

**Authors:** Dechuan Kong, Hao Pan, Huanyu Wu, Jian Chen

**Affiliations:** 1 Department of Communicable Diseases Control and Prevention, Shanghai Municipal Center for Disease Control and Prevention, Shanghai, China; 2 Shanghai Municipal Center for Disease Control and Prevention, Shanghai, China

**Keywords:** artificial intelligence, global public health, infectious diseases, social science, vaccine

## Abstract

The frequency of emerging infectious diseases have exposed the limitations of traditional response models, which are increasingly inadequate for contemporary prevention and control needs. History has repeatedly demonstrated the necessity of multi-disciplinary collaboration. Advances in multiple fields and technological revolutions now provide new tools to address these challenges. “Infectious Diseases+” (ID+) is an interdisciplinary integration concept that centers on infectious disease prevention and control. We introduce ID+ as an innovative paradigm for next-generation epidemic control, featuring: (a) three theoretical breakthroughs vs. conventional methods, (b) cross-disciplinary applications from AI-driven prediction to vaccine equity governance, and (c) validated pediatric use cases. Future scaling pathways are also discussed.

## 1 Introduction

Over the past three decades, emerging infectious diseases such as SARS, H5N1 avian influenza, MERS, Ebola, and COVID-19 have continued to emerge globally, with major outbreaks occurring every 4–5 years on average (1–6). Additionally, climate change is reshaping the geographic distribution of these diseases (7–10). As an example, the habitable range of Aedes mosquitoes, vectors of dengue fever, has expanded in European (11–13). Furthermore, the thawing of permafrost may release unknown pathogens (14–16).

This increased frequency of emerging infectious diseases have exposed the limitations of traditional response models, which are increasingly inadequate for contemporary prevention and control needs. Critical shortcoming with conventional surveillance diagnosis, treatment and vaccine/drug development were revealed during COVID-19. For example, traditional surveillance systems lacked sufficient sensitivity and intelligence, failing to meet the demands of early detection and large-scale emergency response (6, 17). Similarly, vaccine development cycles could not keep pace with the rapid transmission and mutation of viruses (18–20).

History has repeatedly demonstrated the necessity of multi-disciplinary collaboration. Rapid advances across multiple disciplines, alongside revolutionary technological breakthroughs, are now empowering us with novel means to overcome these challenges. Big data analytics, artificial intelligence, nanotechnology, mRNA vaccine technology, CRISPR gene editing, have opened new frontiers in infectious disease control. The multi-point trigger and intelligent early warning surveillance system recently launched by China's National Disease Control and Prevention Administration exemplifies the practical application of the systems like “Infectious Diseases+” (ID+) concept (21, 22). Moreover, the ID+ approach serves as an essential methodology for implementing the One Health framework at the operational level (23–27). The prevention and control of pediatric infectious diseases requires the integrated application of ID+, with its core rationale lying in the unique characteristics of the pediatric population and the complexity of infectious disease management. Undoubtedly, ID+ represents the most critical characteristic of infectious disease prevention and control in the new era.

## 2 The ID+ concept

ID+ is an innovative interdisciplinary integration concept that centers on infectious disease prevention and control. It aims to establish a strategic framework for a comprehensive “prediction > early warning > prevention > treatment” system through interdisciplinary collaboration, technological empowerment, and global governance enhancement. One Health and ID+ are both integrative concepts, but they differ significantly in their scope, core philosophy, and primary focus. ID+ can be a powerful application and a component of the broader One Health philosophy within the specific context of infectious diseases. One Health provides the overarching ethical and theoretical framework for ID + . Our paper marks the global introduction of ID+ concept, whose core philosophy lies in breaking down disciplinary barriers and transforming infectious disease prevention and control from a traditional public health emergency response into a complex integrated systems approach, addressing the addressing the multifaceted challenges of emerging infectious diseases in the modern era.

### 2.1 Differences between ID+ and traditional infectious disease theories

Compared with traditional infectious disease theories, ID+ differs in academic science, response speed, decision making, intervention tool, data flow, governance model, precision level, and cost efficiency. For details, see Table 1.

**Table 1:** Comparison between traditional and next-Gen infectious diseases paradigms.

Dimensions	Traditional infectious diseases	ID+ Approach
Academic Science	Medicine/Epidemiology dominated	Medicine + (AI/Climatology/ Social Science/ others) integration
Response Speed	Reactive (post-outbreak)	Proactive (pre-outbreak interception)
Decision Making	Limited case data	Real-time multisource data fusion (genomic, climate, behavioral, et al)
Intervention Tool	Vaccines + Quarantine + Treatment	Vaccines + AI prediction + Environmental modification + Behavioral nudges
Data Flow	Weekly/Monthly reports	Real-time streaming analytics
Governance Model	Government-led vertical command	Distributed autonomous networks
Precision Level	Population-level measures	Individualized risk profiling
Cost Efficiency	High marginal costs	Declining marginal costs

### 2.2 The three core dimensions and theoretical innovations of ID+

**Figure 1:**
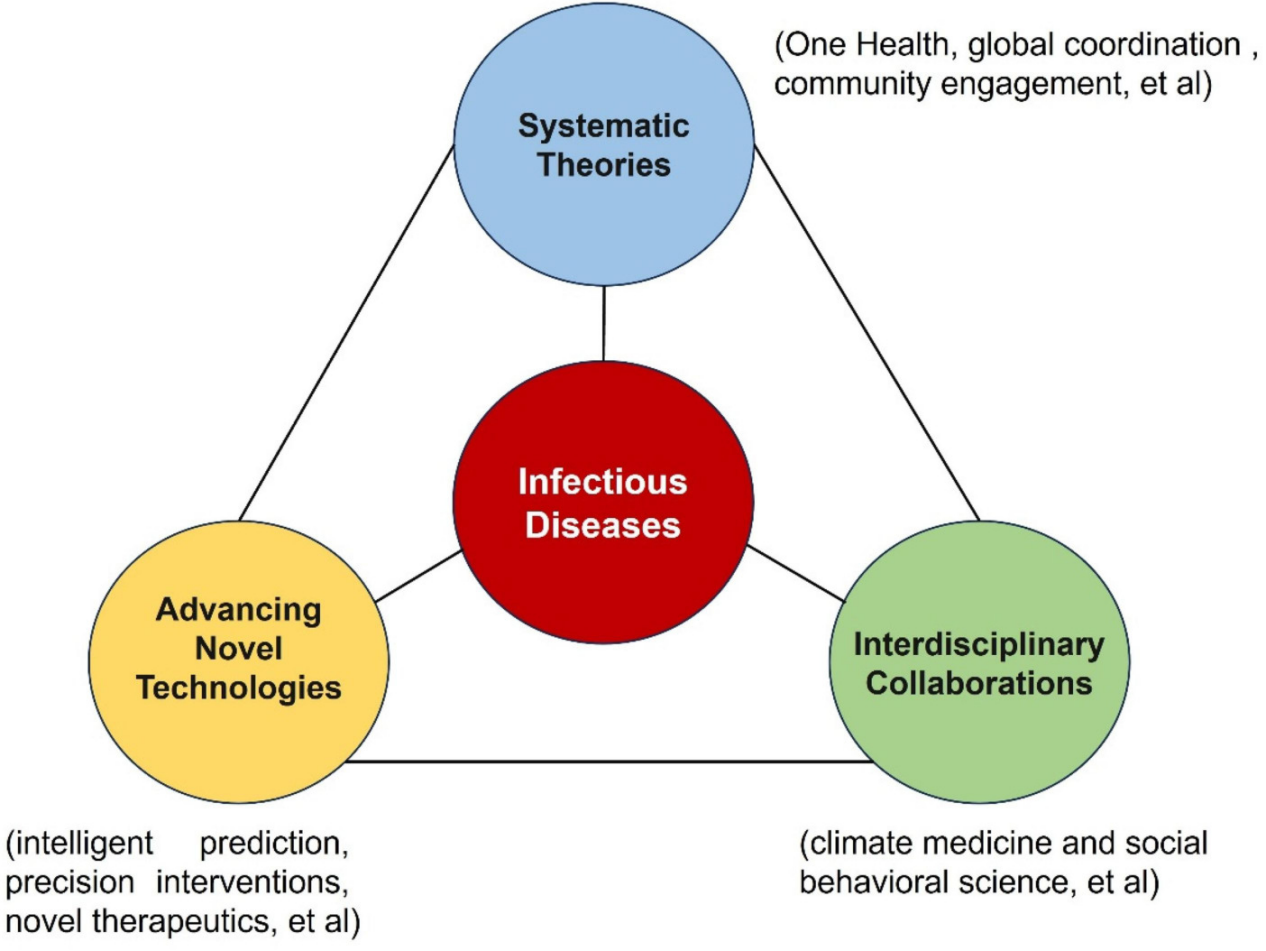
The schematic diagram of three core dimensions and theoretical innovations of ID+. The circles in different colors represent the core of infectious diseases in ID+ and the three introduced elements (systematic theories, advancing novel technologies, and interdisciplinary collaborations). The solid lines indicate the extensive connections and dynamically changing nature between the infectious disease core and each element.

The three core dimensions of ID+ are reflected in advancing novel technologies (intelligent prediction, precision interventions, novel therapeutics, et al.), interdisciplinary collaborations (climate medicine and social behavioral science, et al.), and systematic theories (One Health, global coordination, community engagement, et al.). The innovation of ID+ lies in the application of complexity science (Figure 1). Traditional prevention/control theories of infectious diseases collide with these new elements, selectively assimilating their merits to form ID+—an ecosystem of interconnected, co-adaptive, and perpetually evolving components.

## 3 Core domains of ID+

**Figure 2:**
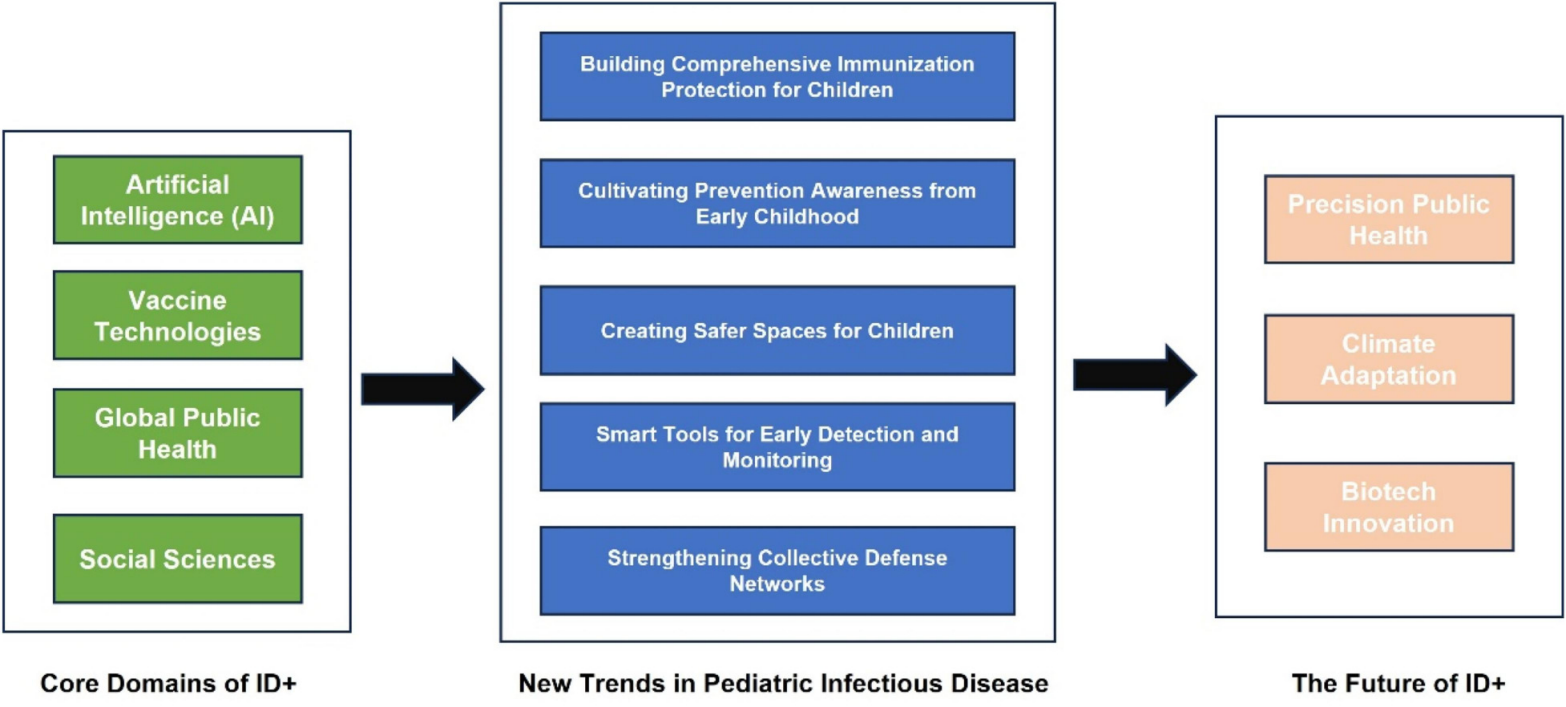
The core components of ID+, Its advancements in pediatric infectious diseases, and future trends.

The core domains of ID+ primarily encompass Artificial Intelligence (AI), Therapeutic Interventions, Global Public Health, and Social Sciences (Figure 2).

### 3.1 Integration of infectious diseases and artificial intelligence (AI)

The ID+ AI approach demonstrates transformative potential across multiple domains: AI-powered epidemic prediction models (e.g., influenza trend analysis via big data) (28–32), rapid pathogen genome sequencing and variant tracking (exemplified by AlphaFold's viral protein structure prediction) (33–37), and intelligent diagnostics (such as AI-based imaging recognition for tuberculosis/malaria) (38–42). Notable implementations include Google DeepMind's breakthrough in forecasting SARS-CoV-2 spike protein structures (43–46) and BlueDot's AI-driven early warning system that detected COVID-19 spread 9 days before WHO alerts (47–49). This synergy enables high-speed real-time analytics (Nanopore Sequencing, Whole Genome Sequencing, Pattern Recognition and Classification Framework using XGBoost and Decision Trees) (50–52) and precision public health (AI-powered screening, Genomic and environmental integration, Personalized Interventions, Pandemic and Outbreak Management) (53–60), fundamentally accelerating the outbreak response paradigm from months to hours.

### 3.2 Integration of infectious diseases and vaccines/therapeutic interventions

The ID+ Vaccines/Therapeutic Interventions paradigm is revolutionizing disease prevention through cutting-edge platforms: mRNA vaccines (exemplified by Moderna/BioNTech's COVID-19 vaccines achieving highest efficacy) (61), nanoparticle vaccines (e.g., malaria/HIV candidates inducing robust T-cell responses) (62, 63), and universal vaccines (broad-spectrum protection against influenza/coronaviruses) (63). This field is transitioning from traditional passive immunization to proactive preventive/therapeutic strategies, as evidenced by cancer vaccines (64). Current innovations enable faster development cycles (COVID-19 vaccines in 9 months vs. historical 10 years) (65–68) and cross-variant protection (nanoparticle flu vaccines covering 20+ strains) (69–71). The future will see vaccines assuming a larger role, showcasing an evolution from prevention to therapy and from infectious to chronic diseases.

### 3.3 Integration of infectious diseases and global public health

The ID+ Global Public Health framework represents a transformative shift in pandemic preparedness through two key innovations: One Health integration (bridging human-animal-environment health monitoring, as seen in cross-species H5N1 surveillance) (72, 73) and global pathogen surveillance networks (exemplified by WHO's Pandemic Accord establishing data-sharing protocols) (74–76). However, this approach faces critical challenges, particularly vaccine equity gaps—during COVID-19, “vaccine nationalism” resulted in 63% of the population of high-income countries were fully vaccinated, compared to just 1.4% of low-income countries (77). Advanced technology platforms (e.g., mRNA vaccines) were predominantly concentrated in wealthier regions, while the introduction and evaluation of other platforms lagged (78). Enhancing the thermostability of vaccines is a critical step toward promoting global vaccine equity. It directly determines whether vaccines can safely and efficiently reach remote and resource-limited areas with unstable electricity and inadequate cold chain infrastructure (79). Modern solutions include GISAID's genomic data platform (accelerating variant analysis) (80–82) and COVAX's allocation mechanism, though systemic reforms remain imperative to achieve the target of 100-day pandemic vaccine deployment for all nations (83–85).

### 3.4 Integration of infectious diseases and social sciences

Non-Pharmaceutical Interventions（NPIs）are actions, outside of vaccines and antiviral drugs, that individuals and communities can take to help slow the spread of respiratory infectious diseases like COVID-19, influenza, and others. They are often the first line of defense, especially when a pathogen is new and no specific pharmaceutical treatments are available. NPIs are typically categorized by the level at which they are implemented: personal, community, and environmental, such as face masks, isolation, quarantine, social distancing. The ID+ Social Sciences approach critically examines behavioral dimensions of outbreaks through two pivotal research streams: misinformation dynamics (where social media algorithms amplified vaccine hesitancy during COVID-19) (86–88) and policy compliance psychology (lockdown adherence correlates with trust in institutions) (89–92) This interdisciplinary field employs computational social science methods which revealed anti-vaccine narratives spread faster than factual content (93–95), while behavioral experiments demonstrate that “nudge”-based interventions (e.g., vaccination lotteries) can increase compliance (96–98) Emerging tools include AI-powered infodemic monitoring (WHO's EPI-WIN system) and cultural risk mapping predicting regional resistance to containment measures (99, 100). Using social media data and machine learning techniques, the preeminent roles parks and greenspaces play during the pandemic and guides a new direction in future urban planning requirements (101).

## 4 New trends in pediatric infectious disease prevention and control in the Era of ID+

New trends in pediatric infectious disease prevention and control under the ID+ framework encompass: (1) ID+ vaccination strategies, (2) ID+ health education, (3) ID+ environmental control, (4) ID+ digital surveillance technologies, and (5) ID+ family-community integrated interventions (Figure 2).

### 4.1 ID+ vaccination: building comprehensive immunization protection for children

The ID+ approach can revolutionize childhood vaccination by integrating advanced technologies and systematic strategies. It advocates for lifelong immunization planning, ensuring vaccine coverage from infancy through adolescence. By developing multivalent combination vaccines (102–105), the number of required injections can be reduced, improving compliance and minimizing discomfort for young children. Big data analytics further enhance vaccination programs by tracking immunization records in real time, identifying missed doses, and enabling targeted catch-up campaigns (106, 107). Mobile health platforms can send automated reminders to parents, while AI-powered systems predict regional outbreaks, allowing preemptive vaccination drives in high-risk areas (108, 109). This data-driven, precision-based model ensures that no child is left unprotected due to logistical or informational gaps.

### 4.2 ID+ health education: cultivating prevention awareness from early childhood

Effective health education is crucial in empowering children to understand and prevent infectious diseases. The ID+ framework promotes age-appropriate educational materials, using animated videos, interactive storybooks, and augmented reality (AR) tools to teach hygiene practices (110–114). Schools and kindergartens integrate handwashing drills, respiratory etiquette, and disease prevention modules into daily routines. Gamification techniques—such as reward-based hygiene apps—make learning engaging, encouraging children to adopt healthy habits voluntarily (115). Teacher training programs ensure educators can deliver accurate, engaging lessons on infectious disease risks. By embedding health literacy early, this approach fosters generations that are likely to me more understanding of the necessity for future public health interventions.

### 4.3 ID+ environmental control: creating safer spaces for children

Children are particularly vulnerable to pathogens in crowded environments like schools and daycare centers. ID+ advocates for enhanced sanitation protocols, including automated disinfection systems, antimicrobial surfaces, and improved ventilation with HEPA filters. Real-time air quality monitors can detect pathogens, triggering instant sterilization in high-risk zones (116–118). Playgrounds and classrooms are redesigned to minimize high-touch surfaces, while UV-C light robots disinfect spaces overnight. Additionally, “green hygiene” solutions—such as plant-based disinfectants—reduce chemical exposure for young children (119, 120). These innovations transform child-centric spaces into low-risk zones, significantly cutting transmission rates of flu, RSV, and other common infections.

### 4.4 ID+ digital technology: smart tools for early detection and monitoring

Digital advancements underpin the ID+ strategy for pediatric care. Wearable devices (e.g., smart thermometers or biosensor patches) track children's vital signs, flagging early symptoms like fever or irregular heart rates (121–123) AI-driven outbreak prediction systems analyze school absenteeism data and online search trends to detect anomalies (124, 125). Electronic health records (EHRs) centralize medical histories, allowing seamless coordination between parents, schools, and doctors (126). Telemedicine platforms enable remote consultations, reducing unnecessary hospital visits. Blockchain technology secures data privacy while ensuring transparent outbreak tracking (127). Together, these tools create a real-time health shield for children, enabling swift responses to potential threats.

### 4.5 ID+ family and community: strengthening collective defense networks

The final pillar of ID+ focuses on community-driven protection. Parents receive training via workshops and mobile apps to recognize symptoms, administer basic care, and implement home quarantine protocols. Neighborhood “buddy systems” connect families for resource sharing (e.g., delivering medicines or groceries during outbreaks) (128, 129). Schools and clinics collaborate through joint drills (e.g., simulating measles containment), while social workers identify at-risk households. Local governments incentivize participation through “healthy community certifications” for districts achieving high vaccination rates (130, 131). By weaving families, schools, and medical institutions into a unified prevention web, the model ensures no child falls through the cracks—turning communities into active defenders against infectious diseases.

### 4.6 Implementation of the ID+ framework for respiratory syncytial virus (RSV) outbreak management in daycare centers

Respiratory Syncytial Virus (RSV) represents a significant and recurrent public health challenge, particularly in daycare settings where close-contact environments facilitate its rapid transmission (132). RSV outbreaks not only cause widespread illness among young children, but also lead to substantial operational disruptions for childcare centers and force parents to miss work. The ID+ Framework offers a structured, multi-phase approach for outbreak management that is particularly suited to the dynamic and high-stakes environment of a daycare center. This framework guides responders from initial identification through to post-outbreak analysis, ensuring a comprehensive and systematic response.

#### 4.6.1 Investigate and detect

The system employs an intelligent, multi-source surveillance mechanism that integrates environmental risk prediction models (133), wastewater-based monitoring (134), the Smart Wearable-Based System for Monitoring Student Symptoms (135), and Saliva-Based rapid molecular testing (136).

#### 4.6.2 Decisive interventions

Infection control and resource allocation strategies incorporate advanced technologies such as robotic disinfection (137), proactive immunoprophylaxis (138), assessment Tool for Respiratory Syncytial Virus Infection in Infants (139).

#### 4.6.3 Disseminate & integrate

The framework strengthens coordinated response and public communication through community-based home monitoring (140), targeted health education (141).

In summary, managing an RSV outbreak in a daycare setting is a complex endeavor. By adopting the proactive, phased, and systematic ID+ Framework, daycare administrators and public health responders can shift from a reactive posture to one of controlled management. This approach not only enhances the effectiveness of outbreak containment but also builds the center's resilience against future infectious disease threats.

## 5 The future of ID+: precision, climate adaptation, and biotech innovation

The ID+ paradigm is evolving toward precision public health, where real-time genomic sequencing integrated with digital contact tracing (like China's Health Code system) enables instant outbreak containment (142). Meanwhile, climate change is reshaping disease patterns, as warming temperatures expand mosquito habitats northward, bringing malaria, dengue and chikungunya to new regions while potentially awakening ancient pathogens from thawing permafrost (143). Synthetic biology presents both unprecedented risks—including engineered biothreats like synthetic smallpox—and revolutionary solutions (144, 145), from CRISPR-modified mosquitoes that block malaria transmission to designer probiotic “shield microbes” that outcompete pathogens (146, 147). This trifecta of digital epidemiology, climate resilience, and genetic engineering will define the next generation of pandemic preparedness, demanding equally sophisticated global governance frameworks to safely harness these transformative technologies (Figure 2).

The future development of ID+ faces severe ethical challenges. The core issue lies in the fact that highly integrated personal data may lead to unprecedented risks of privacy breaches and surveillance; embedded algorithmic biases could solidify social discrimination and systematically exclude certain groups. We must proactively establish ethical and legal frameworks to ensure that technological advancement doesn't come at the cost of compromising human privacy, fairness, and security.
